# Evaluation of Patient-Facing Mobile Apps to Support Physiotherapy Care: Systematic Review

**DOI:** 10.2196/55003

**Published:** 2024-03-04

**Authors:** Mark Merolli, Jill J Francis, Patrick Vallance, Kim L Bennell, Peter Malliaras, Rana S Hinman

**Affiliations:** 1 Centre for Health, Exercise & Sports Medicine Department of Physiotherapy, School of Health Sciences The University of Melbourne Melbourne Australia; 2 Centre for Digital Transformation of Health The University of Melbourne Melbourne Australia; 3 School of Health Sciences The University of Melbourne Melbourne Australia; 4 Department of Physiotherapy, Podiatry, Prosthetics and Orthotics School of Allied Health, Human Service and Sport La Trobe University Melbourne Australia

**Keywords:** physiotherapy, physical therapy, digital health intervention, mobile app, behavior change technique, behavior change, exercise, systematic review, quality, rehabilitation, BCT, mHealth, mobile health, app, apps, physical activity, fitness, synthesis, syntheses, review methods, review methodology, search, searches, searching, systematic, mobile phone

## Abstract

**Background:**

Mobile health interventions delivered through mobile apps are increasingly used in physiotherapy care. This may be because of the potential of apps to facilitate changes in behavior, which is central to the aims of care delivered by physiotherapists. A benefit of using apps is their ability to incorporate behavior change techniques (BCTs) that can optimize the effectiveness of physiotherapeutic interventions. Research continues to suggest that despite their importance, behavior change strategies are often missing in patient management. Evaluating mobile apps that physiotherapists can use to drive behavior change may inform clinical practice and potentially improve patient outcomes. Examining the quality of apps and exploring their key features that can support behavior change and physiotherapy care are important aspects of such an evaluation.

**Objective:**

The primary aim of this study was to describe the range of mobile apps in app stores that are intended for use by patients to support physiotherapy care. The secondary aims were to assess app quality, BCTs, and their behavior change potential.

**Methods:**

A systematic review of mobile apps in app stores was undertaken. The Apple App Store and Google Play were searched using a 2-step search strategy, using terms relevant to the physiotherapy discipline. Strict inclusion and exclusion criteria were applied: apps had to be intended for use by patients and be self-contained (or stand-alone) without the requirement to be used in conjunction with a partner wearable device or another plugin. Included apps were coded for BCTs using the Behavior Change Technique Taxonomy version 1. App quality was assessed using the Mobile App Rating Scale, and the App Behavior Change Scale was used to assess the app’s potential to change behavior.

**Results:**

In total, 1240 apps were screened, and 35 were included. Of these 35 apps, 22 (63%) were available on both the Apple App Store and Google Play platforms. In total, 24 (69%) were general in their focus (eg, not condition-specific), with the remaining 11 (31%) being more specific (eg, knee rehabilitation and pelvic floor training). The mean app quality score (Mobile App Rating Scale) was 3.7 (SD 0.4) of 5 (range 2.8-4.5). The mean number of BCTs identified per app was 8.5 (SD 3.6). BCTs most frequently included in the apps were instruction on how to perform a behavior (n=32), action planning (n=30), and self-monitoring of behavior (n=28). The mean behavior change potential score (App Behavior Change Scale) was 8.5 (SD 3.1) of 21 (range 3-15).

**Conclusions:**

Mobile apps available to support patient care received from a physiotherapist are of variable quality. Although they contain some BCTs, the potential for behavior change varied widely across apps.

**International Registered Report Identifier (IRRID):**

RR2-10.2196/29047

## Introduction

### Background

Digital health in physiotherapy is an emergent area. It continues to gather speed with its use in both clinical practice and research growing exponentially [1-3]. Many digital modalities, with varied functions, are relevant to physiotherapy practice; these might include the delivery of timely digital patient information and resources through websites and patient portals [4], digital patient assessment using connected Bluetooth and wireless-enabled devices [5], digital models of care (telehealth) supported by video-based consultation [6-8], remote monitoring of patient status through wearables [9,10], and mobile health (mHealth) apps used to prescribe, monitor, and support home exercise programs [1,11].

Mobile apps are one type of digital health modality of particular interest because of the ubiquity of smartphone use and their ability to deliver digital therapeutics [12-14]. They are relatively inexpensive and thus scalable [15]. They are also worthy of scientific attention within the physiotherapy community because of their ability to support interventions targeting several aspects of behavior change, otherwise known as behavior change techniques (BCTs) [16-19]. Facilitating positive behavior change in people with health conditions is often central to the management approach of physiotherapists, aimed at deriving the best possible therapeutic benefit from an intervention. For example, behavior change may be required for a person to follow physiotherapist advice regarding activity pacing or to adhere to exercise and physical activity recommendations [1,16,19,20]. BCTs can be thought of as the components of an intervention that regulate the behavior by altering cause and effect [21]. As defined by Michie and Johnston [22], they may be thought of as the “active ingredients” that facilitate intended behaviors. Apps have the ability to incorporate and deliver numerous BCTs, which can mediate a behavioral target of physiotherapy care [23-25].

The app space continues to grow exponentially, with well over 300,000 health apps now available and over 200 health apps added to major app stores daily [18]. Concurrently, despite a growing evidence base regarding the role of mobile apps in physiotherapy contexts, there remains a dearth of high-quality research using validated and rigorous scientific methods assessing the quality of physiotherapy-specific mobile apps and their behavior change aspects (eg, BCTs used and behavior change potential) [11,25].

### Objectives

The primary study objective was to describe mobile apps, intended for use by patients, that can support physiotherapy care. The secondary objective was to evaluate app quality [26] and any BCTs contained within them [21], and to evaluate the behavior change potential of the apps [24].

## Methods

### Study Design

The detailed protocol for this systematic review was previously published [25]. As this study is a systematic review of apps (in app stores) and does not involve human participants, ethics approval was not required. The review followed the PRISMA (Preferred Reporting Items for Systematic Reviews and Meta-Analyses) statement’s systematic review reporting principles, with minor adaptations as relevant for our search of app stores rather than research literature [27]. This method is common and adopted in other similar studies [18,28,29] (Multimedia Appendix 1) [30].

### Search Strategy

#### Overview

A 2-phase search strategy was used to search the popular app stores (Apple App Store and Google Play), which was in line with other rigorous systematic reviews of health apps in app stores [29]. App store search strategy, keywords used, and steps can be seen in Textbox 1 [25]. Initial search was conducted in March 2021 and rerun in February 2023 to ensure further up-to-date coverage. To maintain the feasibility of the search, each search term combination was limited to the first 100 apps retrieved. This was also done to maintain the relevance of apps retrieved, which diminishes as the end of the search list is refreshed [28].

**Search strategy.**

**Step 1**

“physiotherapy,” “physio,” “physical therapy,” “physiotherapist,” “physical therapist.”

**Step 2**

“physiotherapy,” “physio,” “physical therapy,” “physiotherapist,” “physical therapist.”

and

“assessment,” “diagnosis,” “digital,” “eHealth,” “evaluation,” “examination,” “exercise,” “health promotion,” “intervention,” “physical activity,” “plan,” “care,” “prevention,” “rehabilitation,” “screening,” “pain,” “self-management,” “treatment,” “support,” “adherence.”

Additionally, the websites of the top 10 member organizations of World Physiotherapy (the peak international physiotherapy body) based on number of members were searched for recommendation or endorsement of any specific apps that met our criteria. Only websites in English and with the relevant section not behind a paywall were searched.

#### Selection Process

The search was performed independently by 2 reviewers (MM and PV). The same reviewers independently screened the apps using 1 Apple and 1 Android device each. Screening was based on information in the respective app stores, including app title, description, and screenshots [31]. Apps eligible for full analysis were downloaded onto the devices for review by each reviewer independently, and any disagreements were resolved by discussion. A third reviewer (PM) was flagged as a mediator to resolve any nonconsensus, but this was not required [28].

### Data Extraction

#### Overview

The 2 reviewers (MM and PV) independently extracted descriptive data about each app from the app stores, within the apps themselves, or from official websites of the apps (if readily apparent from information in the app store). All extracted data were computed in Microsoft Excel (Microsoft Corp). A full list of extracted descriptive characteristics is shown in Multimedia Appendix 2 [25].

Both reviewers (MM and PV) independently engaged with all of the app functions for a minimum of 10 minutes on each device, for familiarization. This allowed each reviewer to independently code and score the app quality, BCTs, and their behavior change potential.

#### Mobile App Quality

To appraise the quality of included apps, the 23-item Mobile App Rating Scale (MARS) was used [26]. MARS was used because of its reliability, simplicity, and wide applicability in mHealth research [26,32]. Its primary domains are engagement, functionality, aesthetics, and information, as well as a subjective rating of quality. Each domain has questions coded on a 5-point Likert scale (ranging from 1=inadequate to 5=excellent). Mean scores are calculated for each of the 4 domains, which are tallied and divided by the number of domains to produce an overall mean quality score out of 5. Mean scoring is used instead of total scores because individual items can be rated as “not applicable” [26].

Calculating the mean scores of the engagement, functionality, aesthetics, and information quality objective subscales and an overall mean app quality total score is how the MARS is scored. Mean scores instead of total scores are used because an item can be rated as “not applicable.”

Both reviewers (MM and PV) completed MARS training before scoring each app independently. Any disagreements were rectified via discussion. A third reviewer (PM) was flagged as a mediator to resolve any nonconsensus, but this was not required.

#### Coding BCTs

Both reviewers (MM and PV) underwent and received certification in coding BCTs using the Behavior Change Technique Taxonomy version 1 (BCTTv1). This was to increase their competence in identifying and assessing BCTs and to improve coding agreement [33]. Based on the minimum of 10 minutes they engaged with app functions, each reviewer independently coded the BCTs incorporated in each app using the BCTTv1, a framework of 93 BCTs created for investigating behavior change interventions using valid and reliable methods [21]. Furthermore, the BCTs in the BCTTv1 are further arranged into 16 clusters, each including similar BCTs [21]. This clustering was helpful for coders when developing a novel codebook (Multimedia Appendix 3), as it helped clarify coding decisions when examining the behavior change potential of included apps [25]. Any disagreements were discussed to achieve consensus. Where consensus was not able to be achieved, a third reviewer (JJF; who is a behavior change expert and one of the original developers of the BCTTv1) helped resolve any disagreements.

#### Behavior Change Potential

The App Behavior Change Scale (ABACUS) was used to evaluate the behavior change “potential” of each app, which is a scale of 21 items [24]. Apps are scored by identifying any of the 21 items and summing these item scores to give a score out of 21. The ABACUS focuses on 4 BCT clusters: knowledge and information, goals and planning, feedback and monitoring, and actions [24]. Using clustered BCTs identified, we developed the aforementioned novel coding rulebook to support reporting, and the 2 reviewers (MM and PV) scored behavior change potential together using the ABACUS. Any disagreements were discussed at the time, and a third reviewer (JJF) decided in the case of nonconsensus.

### Data Synthesis

App characteristics are reported as descriptive and categorical data and a proportion (percentage), including app name, data privacy transparency (yes or no), companion app (yes or no), platform (Apple App Store, Google Play, or both), focus of the app (specific condition or region or whether more general), physiotherapy specialty (eg, musculoskeletal, pelvic health, and pediatric), target behaviors, simplified target behaviors, country of origin, developer qualifications (not clear or health care professional [HCP] or non-HCP), app version number, payment method (free or in-app purchases or one-off payment), and cost. Overall mean (SD) and individual domain scores are presented for MARS and ABACUS scores, as well as total BCT identification frequency and BCTs coded per app.

## Results

### Search

**Figure 1:**
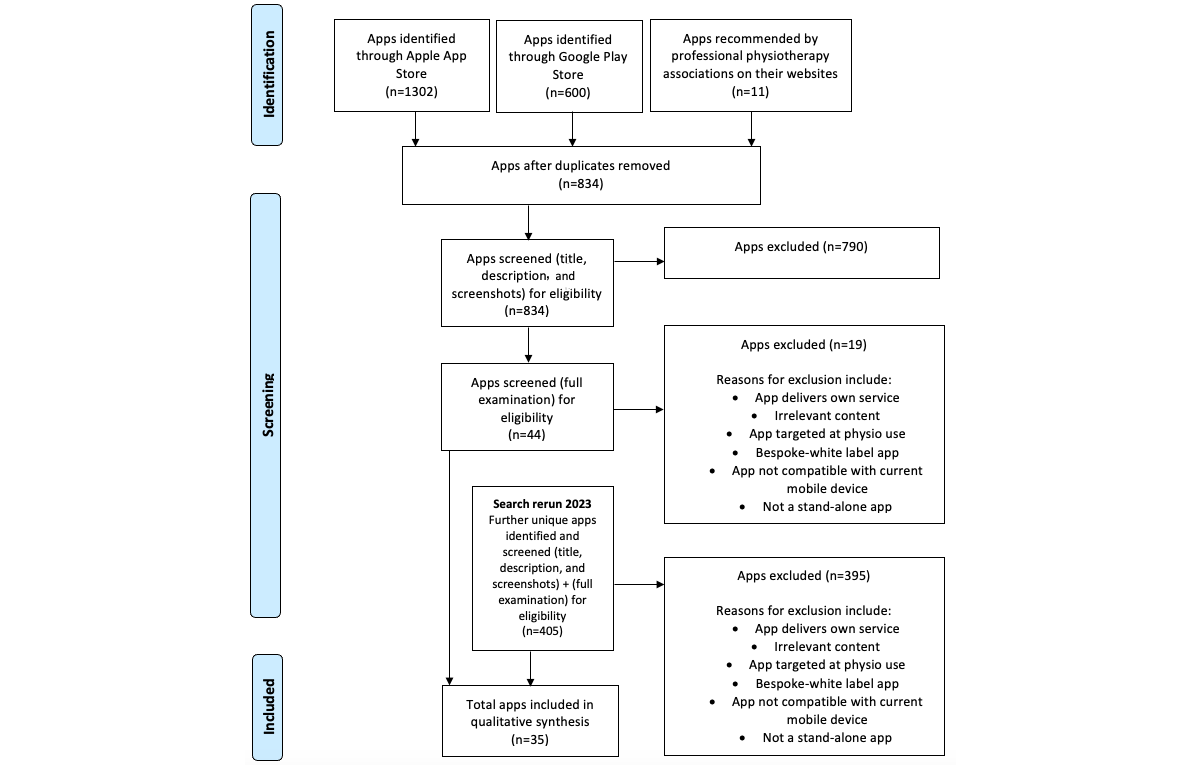
PRISMA (Preferred Reporting Items for Systematic Reviews and Meta-Analyses) flow diagram.

Our initial search identified a total of 1913 apps (Figure 1). This included 1302 unique apps from the Apple App Store, 600 apps from the Google Play Store, and a further 11 apps identified on the websites of professional physiotherapy associations. Overall, 834 were left after duplicates were removed. Following screening of app name, description, and screenshots, a further 790 were excluded. In total, 44 apps were screened fully, of which 19 were excluded. Reasons for exclusion included non-English language, costing more than Aus $10 (a currency exchange rate of Aus $1=US $0.72 is applicable), delivering its own service, irrelevant content, targeted at physiotherapist (clinician use) rather than the patient, being a bespoke or white-labeled app (ie, standard architectures licensed to a business or brand for private use), incompatibility with a current mobile device, and not a stand-alone app. This left 25 included apps for data extraction. Search update was rerun in February 2023, yielding an additional 405 unique apps. Following screening, this yielded a further 10 included apps for data extraction. Thus, a total of 35 apps were examined.

### Descriptive Characteristics of Included Apps

App characteristics are described in Multimedia Appendix 2. Overall, 22 (63%) apps were available on both Apple App Store and Google Play platforms, and 12 (34%) only on Apple App Store. Only 1 (3%) app was solely available on Google Play. Country of origin was clear for 24 (69%) apps, with 6 apps originating from the United Kingdom, 6 from the United States, and 5 from Australia. Regarding price, 26 (74%) were free to download, and 9 had a one-off payment price ranging from Aus $1.49 to $9.99. Additionally, 10 apps offered in-app purchases (including subscriptions). Just under half of the apps (n=16, 46%) were not clear about the developer’s qualifications, a further 17 (49%) were developed by HCPs (ie, physiotherapists, orthopedic surgeons, exercise physiologists, and chiropractors), and 2 (6%) were developed by non-HCPs. Overall, 15 (43%) apps had an obvious physiotherapist companion feature (ie, dashboard, desktop software, or precise login for therapists to access and engage with patients), and 31 (89%) apps had information about privacy and security in the app store. The focus of most apps was general in nature (eg, not condition-specific; n=24, 69%), while 11 (31%) had a specific target (eg, knee rehabilitation and pelvic floor training). This included 3 apps focusing on pelvic health and 1 app each focusing on wrist or hand, knee, hemophilia, core stability, wheelchair-bound individuals, cerebral palsy, osteoarthritis, and back pain.

### Assessment of Quality (MARS)

Table 1 shows app quality scores using the MARS. Individual app quality scores ranged from 2.8 to 4.5, with a mean score of 3.7 (SD 0.4) of a maximum of 5. The apps with the highest MARS scores were Squeezy: CF (4.5), Squeezy (4.4), AllyCare (4.3), Squeezy for Men (4.2), and TeleHab (4.2). Of the appraised MARS domains, “aesthetics” (mean 3.9, SD 0.6) and “functionality” (mean 3.8, SD 0.5) were the highest scoring sections, while “subjective app quality” (mean 3.4, SD 0.8) and “engagement” (mean 3.4, SD 0.5) were the lowest. To the best of our knowledge, only 4 (11%) apps met criterion 19 (has the app been trialed or tested and published in scientific literature), including ReHand, Hand Rehabilitation, Squeezy, Embodia, and PhysiApp. Only 1 (2.9%) app (ReHand, Hand Rehabilitation) has been evaluated in a randomized controlled trial (RCT), showing positive outcomes for physiotherapy patients [34,35].

**Table 1:** Mobile App Rating Scale scoring.

App	Section A: engagement	Section B: functionality	Section C: aesthetics	Section D: information	Mean app quality score (section A to D)	Section E: subjective app quality
A Rehab Diary	3.6	3.0	2.7	3.2	3.1	2.5
AllyCare	4.4	4.0	4.7	4.2	4.3	4.5
Back Pain Diary	3.4	4.0	3.7	2.8	3.5	2.5
BlueJay Engage - Patient	3.8	4.0	4.3	4.0	4.0	4.0
ComplexCore	2.4	4.0	4.0	3.3	3.4	3.0
CP-Fit	3.2	3.5	4.0	3.3	3.5	3.0
Embodia	3.8	4.0	4.3	3.6	3.9	4.0
ExorLive	3.6	3.3	3.0	3.1	3.3	3.0
Extensor	3.4	3.5	3.3	3.3	3.4	3.0
Guided Physio	3.4	4.3	4.3	3.8	4.0	4.0
HaemActive	3.6	3.5	4.7	3.6	3.8	4.0
Home Physio	3.0	4.0	4.3	3.3	3.7	2.5
My Exercise Messages	3.4	4.3	4.0	4.8	4.1	3.5
My Exercise Program	3.6	3.8	4.0	3.8	3.8	3.3
My Injury Exercises	2.8	4.0	4.0	3.5	3.6	3.3
OT App Lite	2.6	3.5	4.0	3.0	3.3	2.0
PhysiApp	3.4	4.3	4.3	3.7	3.9	4.0
Physiotools Trainer	3.0	3.5	3.3	3.3	3.3	3.3
Pocket Physio	2.4	2.5	3.0	3.3	2.8	2.3
PT Timer: Stretch & Exercise	3.0	3.5	3.0	3.2	3.2	2.0
PT-Helper Pro	3.0	3.3	3.3	3.0	3.2	2.5
RecovAware Knee Health Fitness	4.2	3.8	4.3	4.2	4.1	4.0
Rehab Guru Client	3.8	3.8	4.3	3.8	3.9	4.0
ReHand, Hand Rehabilitation	3.6	4.3	4.3	4.1	4.1	4.0
Smart Therapist	3.4	3.5	3.3	3.5	3.4	3.3
Squeezy: CF	4.0	5.0	4.3	4.5	4.5	4.5
Squeezy for Men	3.6	4.8	4.3	4.3	4.2	4.5
Squeezy	4.0	5.0	4.3	4.3	4.4	5.0
Switchback Health	3.2	3.8	3.7	3.5	3.5	3.0
TeleHab	4.0	4.5	4.7	3.7	4.2	4.0
Track Rehab	3.4	3.3	3.3	3.8	3.5	3.0
TrackActive Pro - Patient App	3.4	3.5	3.7	3.8	3.6	3.5
VR steps Home rehabilitation	4.0	3.8	3.7	3.3	3.7	3.3
Wheelchair Exercises	3.0	3.8	4.0	3.5	3.6	3.3
YRMOVE	3.0	3.5	3.0	2.6	3.0	2.3
Mean (SD)	3.4 (0.5)	3.8 (0.5)	3.9 (0.6)	3.6 (0.5)	3.7 (0.4)	3.4 (0.8)

### Behavior Change: Target Behaviors and BCTs

The most frequently observed target behaviors in the apps included recording information about exercise (n=28, 80%), performing therapeutic exercise (general; n=25, 71%), and communicating with a health professional (n=12, 34%; Multimedia Appendix 2). Other observed target behaviors included performing therapeutic exercise (men’s health, women’s health, hand therapy, knee, and perioperative), connecting with a health professional (make an appointment), connecting with friends, general self-care, and postsurgical self-care.

Coded BCTs by frequency can be observed in Multimedia Appendix 4, with a glossary of BCTs presented in Multimedia Appendix 5. The mean number of BCTs identified per app was 8.5 (SD 3.6). The apps with the highest number of unique BCTs were AllyCare (16), My Exercise Messages (14), ReHand, Hand Rehabilitation (14), and PhysiApp, BlueJay Engage, A Rehab Diary, and TeleHab (all with 13 BCTs identified). The most frequently coded BCTs were BCT 4.1 (instruction on how to perform a behavior; coded in n=32, 91% apps), BCT 1.4 (action planning; coded in n=30, 86% apps), BCT 2.3 (self-monitoring of behavior; coded in n=28, 80% apps), BCT 2.2 (feedback on behavior; coded in n=27, 77% apps), BCT 6.1 (demonstration of the behavior; coded in n=27, 77% apps), BCT 7.1 (prompts or cues; coded in n=25, 71% apps), and BCT 9.1 (credible source; coded in n=25, 71% apps).

### Assessment of Behavior Change Potential (ABACUS)

Table 2 shows ABACUS scores for each app. The behavior change potential of included apps was a mean of 8.5 (SD 3.1) of a maximum of 21 (range 3-15). Of the 4 domains assessed by the ABACUS, section 1 (knowledge and information: mean score 3.0, SD 0.9) and section 3 (feedback and monitoring: mean score 2.9, SD 1.6) scored the highest, while section 2 (goals and planning: mean score 0.3, SD 0.8) and section 4 (actions: mean score 2.4, SD 1.0) scored the lowest. The apps (n=21) with the highest ABACUS scores included AllyCare (n=15, 71%), My Exercise Messages (n=14, 67%), ExorLive Go (n=14, 67%), A Rehab Diary (n=13, 62%), and both BlueJay Engage (n=12, 57%) and ReHand, Hand Rehabilitation (n=12, 57%).

**Table 2:** App Behavior Change Scale scoring.

	Section 1: Knowledge and information	Section 2: Goals and planning	Section 3: Feedback and monitoring	Section 4: Actions	Total
A Rehab Diary	3	2	5	3	13
AllyCare	4	2	6	3	15
Back Pain Diary	1	0	3	0	4
BlueJay Engage - Patient	4	1	4	3	12
ComplexCore	3	0	1	2	6
CP-Fit	2	0	3	2	7
Embodia	4	0	4	3	11
ExorLive	4	2	5	3	14
Extensor	3	0	4	2	9
Guided Physio	4	0	2	0	6
HaemActive	4	0	2	3	9
Home Physio	2	0	0	3	5
My Exercise Messages	3	3	4	4	14
My Exercise Program	3	0	3	2	8
My Injury Exercises	3	0	0	1	4
OT App Lite	2	0	0	1	3
PhysiApp	4	0	4	3	11
Physiotools Trainer	2	0	3	3	8
Pocket Physio	4	0	0	3	7
PT Timer: Stretch & Exercise	2	0	3	3	8
PT-Helper Pro	3	0	3	3	9
RecovAware Knee Health Fitness	3	0	3	3	9
Rehab Guru Client	2	0	4	3	9
ReHand, Hand Rehabilitation	3	0	5	4	12
Smart Therapist	2	0	3	2	7
Squeezy: CF	4	0	2	3	9
Squeezy for Men	4	0	2	3	9
Squeezy	4	0	2	3	9
Switchback Health	2	0	4	3	9
TeleHab	4	0	5	2	11
Track Rehab	4	0	4	3	11
TrackActive Pro - Patient App	2	0	3	2	7
VR steps Home rehabilitation	3	0	3	1	7
Wheelchair Exercises	2	0	0	1	3
YRMOVE	2	0	1	1	4
Mean (SD)	3.0 (0.9)	0.3 (0.8)	2.9 (1.6)	2.4 (1.0)	8.5 (3.1)

## Discussion

### Principal Findings

#### Implications

The primary aim of this study was to describe apps, intended for use by patients, that can support physiotherapy care. The secondary aims were to evaluate app quality, examine BCTs they contained, and evaluate behavior change potential. The findings of this study offer valuable insights into the current landscape of apps that may be used to support physiotherapy care and shed light on their ability to facilitate behavior change and potentially improve patient outcomes.

We identified 35 eligible apps, highlighting the popularity and increasing recognition of digital strategies for supplementing physiotherapy care [2,36]. It must also be noted that there is a growing number of apps within the broader exercise medicine and rehabilitation science space that may not have met the specific eligibility criteria for this systematic review but may still be of value in supporting physiotherapy care [1,18]. The rising number of available mobile apps necessitates careful and considered evaluation and selection of apps by clinicians and researchers to ensure that patients are accessing high-quality, effective, and safe apps that are evidence-based [1,11,37,38].

#### App Characteristics

Analysis of the app descriptive characteristics revealed interesting findings. Just under two-thirds of apps were cross-platform compatible (available on both the Google Play Store and Apple App Store). As smartphone ubiquity continues to grow, broader availability may contribute to wider accessibility and increase the uptake of apps to supplement physiotherapy care by both patients and physiotherapists [39]. The physiotherapy profession has recognized the value of mHealth tools to serve under-resourced communities and regions [40]. It is encouraging to note that in terms of price, the majority of the apps included in our review were free to download for patients. It must also be acknowledged that our review only examined apps that cost Aus $10 or less, which is in line with similar studies of apps for the management of arthritis, back pain, and persistent pain [29,31,32,41]. This follows similar research, suggesting that health consumers are less likely to buy health apps costing more than this [42]. However, there may well be more expensive apps available for purchase that were not included in our review. Conversely, while there are many seemingly free-to-download apps, several of the included apps require the physiotherapist to prescribe the patient a program from a companion app or dashboard before they can use it (eg, PhysiApp and TeleHab). In situations like this, the use of apps to support care is contingent on factors such as willingness to engage with digital health, acceptance, and digital health literacy of treating physiotherapists [2,37,38].

Relevant to an evidence-based profession like physiotherapy, the credibility of apps to support care remains an important consideration, and research suggests that this is a central factor in user engagement and satisfaction [43,44]. Less than half of the apps 16 (46%) provided clear information about the qualifications or background of the developers. Similarly, only 17 (49%) apps were clearly developed by health professionals (including physiotherapists). Research suggests that apps developed by or with health professionals may contribute to improved efficacy and safety, outcomes, and evidence-based care delivered by the apps [45]. However, while this may be true, a counterargument stands that while said to be developed by health professionals, it remains unclear what evidence was used in developing these apps.

#### Quality

A key aim of this study was to assess the quality of mobile apps relevant to physiotherapy care. While app quality is somewhat subjective, and measurement scales continue to emerge (eg, the recently released Deakin Health E-technologies Assessment Lab framework) [46], the MARS remains the most used, published, and validated [1,26,29]. Overall mean app quality in this study was 3.7 (SD 0.4), which is similar to previous research validating the MARS [47]. The authors reported a mean health app quality score of 3.74 (SD 0.6) of 5 and considered this as “moderate” quality. Notably, there is no universally accepted threshold for interpreting MARS scores. This suggests that the current landscape of physiotherapy apps has a little way to go in terms of quality. Our MARS data for the domains of “aesthetics” and “functionality” suggested that the included apps generally offer a good user experience and technical performance. These findings align with a quality appraisal of mobile apps for self-management of persistent pain conditions [29] but contrast to the relatively low scores obtained for apps specific to the management of low back pain [32].

Given that the physiotherapy profession is an evidence-based discipline, it is alarming that we found that only 4 (11%) of included apps met MARS criterion 19 (has the app been trialed or tested and published in scientific literature). Further, only 1 (3%; ReHand, Hand Rehabilitation) has been examined in an RCT [34,35,48]. However, the SMS-based precursor, which informed the development of My Exercise Messages, has undergone rigorous RCTs, showing positive patient outcomes [48,49]. An RCT studying the My Exercise Messages app is currently underway, and the protocol has been published [50]. As digital physiotherapy practice evolves, a combination of (1) greater assessment and scrutiny of digital health tools (eg, apps) through standardized validated measures and (2) high-quality well-designed RCTs and systematic reviews will assist in building evidence for and ultimately trust in digital tools that can support physiotherapy care [51-53].

#### Behavior Change

Another aim was to determine the BCTs included in apps and the apps’ potential for behavior change. Facilitating patient behavior change is an important aspect of physiotherapy care that may determine the effectiveness of many physiotherapy interventions [54-56]. Analysis of target behaviors suggested that the apps included in this review most commonly targeted behaviors linked to recording information about exercise, performing therapeutic exercise, and communicating with a health professional. This is encouraging, as these behaviors are often central to the core aims of many physiotherapeutic interventions [55,57]. The most frequently occurring BCTs coded within the included apps were instruction on how to perform a behavior, action planning, self-monitoring of behavior, feedback on behavior, demonstration of the behavior, prompts or cues, and credible source, and these align very well to digital behavior change interventions using apps reported in literature on physical activity in osteoarthritis, various musculoskeletal conditions, and falls prevention exercise [58-60].

The potential for behavior change (based on ABACUS findings) could only be considered modest, given the mean ABACUS score of 8.5/21 (SD 3.1; 41%) across the 35 apps evaluated. This is similar to a recent comprehensive analysis of mobile apps to support behavior change in patients with a chronic disease or multimorbidity. In that study, mean ABACUS score was 8.07/21 (38%) [19]. Given that the developers of the ABACUS [24] have highlighted that the clinical implications of ABACUS score are still to be determined, it is not possible to determine whether the apps included in this study are effective or not at changing behavior or if they are likely to be effective at improving clinical outcomes from physiotherapy care. Future prospective research is required to evaluate if the apps included in our review can change patient behavior over time.

### Limitations

The study has limitations. The review only analyzed apps in the English language, which limits the generalizability of the findings. Importantly, the search strategy had specific and stringent inclusion or exclusion criteria, which may lead to interpretative bias. For instance, apps had to be directly identifiable as being supportive of care between a patient and their physiotherapist. While several apps in app stores may indirectly support physiotherapy (eg, general exercise or informational apps), these were out of scope. Similarly, our search was based on a specified set of keywords, and thus, retrieval may not have captured all relevant apps. Furthermore, some apps originally identified in the search strategy were not able to be trialed for several reasons such as no free trial readily available, not available in the select region, or no response by developers for limited-time access by the study authors. A similar scenario occurred for apps that were subscription-based. In these cases, free trials were examined where possible, which may have not included full app functionality, thus potentially leading to more conservative quality, BCT, and behavioral potential scores. Regarding app quality, this was not appraised by patients themselves in this study. Should this have occurred, a different version of the MARS, the user-MARS, would have been required [61]. It is possible that patients may achieve a different quality rating for the apps. This is an area worthy of future research.

In addition, our protocol involved apps being trialed for a minimum of 10 minutes [25,29]. Vaghefi and Tulu [62] suggest “... most users tend to withdraw from mHealth apps before the end of the first week.” This raises the possibility that engaging with each app for a limited amount of time can make it difficult to get a complete picture of an app, with certain features and BCTs not being immediately apparent. This was observed firsthand in the case of “My Exercise Messages,” which scored 14/21 on the ABACUS in this study but scored 17/21 when rated by its developers who are intimately familiar with all the features of the entire 24-week app program [50]. The discrepancy is likely due to the fact that the more BCTs become apparent, the longer a user engages with the app over 6 months. A similar issue lies in the possibility that important descriptive data about an app may be missed or inaccurate because we relied on limited data sources. For instance, not all the necessary detailed information about an app can be found in the app itself or within its app store description. In select cases, this information may be identifiable if publications about the app exist. However, our protocol was deliberately designed to more closely mimic how a lay user would likely find and interact with an app through an app store. For these reasons, the findings reported in this study may be conservative for some apps, and it is possible that quality and behavior change scores would be higher with proper in-depth use. We thus urge readers to use caution in interpreting our findings.

Finally, this study focused primarily on app descriptive characteristics, quality, BCTs, and behavior change potential. It was not an aim of this review to evaluate the clinical effectiveness of these apps in changing behavior or their efficacy toward improving patient health outcomes.

### Conclusions

Mobile apps available to support patient care received from a physiotherapist are of variable quality and contain relatively few BCTs. The potential for behavior change varied widely across apps. This study has provided the first comprehensive examination of mobile apps specifically supporting the care of patients receiving physiotherapy.

## Appendix

Multimedia Appendix 1 PRISMA (Preferred Reporting Items for Systematic Reviews and Meta-Analyses) checklist.

Multimedia Appendix 2 App descriptive data.

Multimedia Appendix 3 Rulebook to support App Behavior Change Scale coding.

Multimedia Appendix 4 Coded behavior change techniques by frequency.

Multimedia Appendix 5 Coded behavior change techniques and glossary.

## Abbreviations

ABACUS: App Behavior Change Scale
BCT: behavior change technique
BCTTv1: Behavior Change Technique Taxonomy version 1
MARS: Mobile App Rating Scale
mHealth: mobile health
PRISMA: Preferred Reporting Items for Systematic Reviews and Meta-Analyses
RCT: randomized controlled trial

## References

[1] Agnew JMR, Hanratty CE, McVeigh JG, Nugent C, Kerr DP (2022). An investigation into the use of mHealth in musculoskeletal physiotherapy: scoping review. JMIR Rehabil Assist Technol.

[2] Merolli M, Gray K, Choo D, Lawford BJ, Hinman RS (2022). Use, and acceptability, of digital health technologies in musculoskeletal physical therapy: a survey of physical therapists and patients. Musculoskelet Care.

[3] Estel K, Scherer J, Dahl H, Wolber E, Forsat ND, Back DA (2022). Potential of digitalization within physiotherapy: a comparative survey. BMC Health Serv Res.

[4] Slater H, Stinson JN, Jordan JE, Chua J, Low B, Lalloo C, Pham Q, Cafazzo JA, Briggs AM (2020). Evaluation of digital technologies tailored to support young people's self-management of musculoskeletal pain: mixed methods study. J Med Internet Res.

[5] Rose MJ, Costello KE, Eigenbrot S, Torabian K, Kumar D (2022). Inertial measurement units and application for remote health care in hip and knee osteoarthritis: narrative review. JMIR Rehabil Assist Technol.

[6] Hörder H, Nero H, Ignjatovic MM, Kiadaliri A, Lohmander LS, Dahlberg LE, Abbott A (2022). Digitally delivered exercise and education treatment program for low back pain: longitudinal observational cohort study. JMIR Rehabil Assist Technol.

[7] Malliaras P, Merolli M, Williams CM, Caneiro JP, Haines T, Barton C (2021). 'It's not hands-on therapy, so it's very limited': telehealth use and views among allied health clinicians during the coronavirus pandemic. Musculoskelet Sci Pract.

[8] Bennell KL, Lawford BJ, Metcalf B, Mackenzie D, Russell T, van den Berg M, Finnin K, Crowther S, Aiken J, Fleming J, Hinman RS (2021). Physiotherapists and patients report positive experiences overall with telehealth during the COVID-19 pandemic: a mixed-methods study. J Physiother.

[9] Ummels D, Beekman E, Moser A, Braun SM, Beurskens AJ (2020). Patients' experiences with commercially available activity trackers embedded in physiotherapy treatment: a qualitative study. Disabil Rehabil.

[10] Vallati C, Virdis A, Gesi M, Carbonaro N, Tognetti A (2018). ePhysio: a wearables-enabled platform for the remote management of musculoskeletal diseases. Sensors (Basel).

[11] Thompson D, Rattu S, Tower J, Egerton T, Francis J, Merolli M (2023). Mobile app use to support therapeutic exercise for musculoskeletal pain conditions may help improve pain intensity and self-reported physical function: a systematic review. J Physiother.

[12] Arntz A, Weber F, Handgraaf M, Lällä K, Korniloff K, Murtonen KP, Chichaeva J, Kidritsch A, Heller M, Sakellari E, Athanasopoulou C, Lagiou A, Tzonichaki I, Salinas-Bueno I, Martínez-Bueso P, Velasco-Roldán O, Schulz RJ, Grüneberg C (2023). Technologies in home-based digital rehabilitation: scoping review. JMIR Rehabil Assist Technol.

[13] Elgert L, Steiner B, Saalfeld B, Marschollek M, Wolf KH (2021). Health-enabling technologies to assist patients with musculoskeletal shoulder disorders when exercising at home: scoping review. JMIR Rehabil Assist Technol.

[14] Bailey JF, Agarwal V, Zheng P, Smuck M, Fredericson M, Kennedy DJ, Krauss J (2020). Digital care for chronic musculoskeletal pain: 10,000 participant longitudinal cohort study. J Med Internet Res.

[15] Silva BMC, Rodrigues JJPC, de la Torre Díez I, López-Coronado M, Saleem K (2015). Mobile-health: a review of current state in 2015. J Biomed Inform.

[16] Greenstein J, Topp R, Etnoyer-Slaski J, Staelgraeve M, McNulty J (2021). Effect of a mobile health app on adherence to physical health treatment: retrospective analysis. JMIR Rehabil Assist Technol.

[17] Rintala A, Rantalainen R, Kaksonen A, Luomajoki H, Kauranen K (2022). mHealth apps for low back pain self-management: scoping review. JMIR Mhealth Uhealth.

[18] Ryan S, Chasaide NN, O' Hanrahan S, Corcoran D, Caulfield B, Argent R (2022). mHealth apps for musculoskeletal rehabilitation: systematic search in app stores and content analysis. JMIR Rehabil Assist Technol.

[19] Bricca A, Pellegrini A, Zangger G, Ahler J, Jäger M, Skou ST (2022). The quality of health apps and their potential to promote behavior change in patients with a chronic condition or multimorbidity: systematic search in App Store and Google Play. JMIR Mhealth Uhealth.

[20] Chen M, Wu T, Lv M, Chen C, Fang Z, Zeng Z, Qian J, Jiang S, Chen W, Zhang J (2021). Efficacy of mobile health in patients with low back pain: systematic review and meta-analysis of randomized controlled trials. JMIR Mhealth Uhealth.

[21] Michie S, Richardson M, Johnston M, Abraham C, Francis J, Hardeman W, Eccles MP, Cane J, Wood CE (2013). The behavior change technique taxonomy (v1) of 93 hierarchically clustered techniques: building an international consensus for the reporting of behavior change interventions. Ann Behav Med.

[22] Michie S, Johnston M, Gellman MD, Turner JR (2013). Behavior change techniques. Encyclopedia of Behavioral Medicine.

[23] Willett M, Duda J, Gautrey C, Fenton S, Greig C, Rushton A (2017). Effectiveness of behavioural change techniques in physiotherapy interventions to promote physical activity adherence in patients with hip and knee osteoarthritis: a systematic review protocol. BMJ Open.

[24] McKay FH, Slykerman S, Dunn M (2019). The app behavior change scale: creation of a scale to assess the potential of apps to promote behavior change. JMIR Mhealth Uhealth.

[25] Merolli M, Francis JJ, Vallance P, Bennell KL, Malliaras P, Hinman RS (2021). Patient-facing mobile apps to support physiotherapy care: protocol for a systematic review of apps within app stores. JMIR Res Protoc.

[26] Stoyanov SR, Hides L, Kavanagh DJ, Zelenko O, Tjondronegoro D, Mani M (2015). Mobile app rating scale: a new tool for assessing the quality of health mobile apps. JMIR Mhealth Uhealth.

[27] Moher D, Liberati A, Tetzlaff J, Altman DG, PRISMA Group (2009). Preferred reporting items for systematic reviews and meta-analyses: the PRISMA statement. PLoS Med.

[28] Richardson B, Dol J, Rutledge K, Monaghan J, Orovec A, Howie K, Boates T, Smit M, Campbell-Yeo M (2019). Evaluation of mobile apps targeted to parents of infants in the neonatal intensive care unit: systematic app review. JMIR Mhealth Uhealth.

[29] Devan H, Farmery D, Peebles L, Grainger R (2019). Evaluation of self-management support functions in apps for people with persistent pain: systematic review. JMIR Mhealth Uhealth.

[30] Page MJ, McKenzie JE, Bossuyt PM, Boutron I, Hoffmann TC, Mulrow CD, Shamseer L, Tetzlaff JM, Akl EA, Brennan SE, Chou R, Glanville J, Grimshaw JM, Hróbjartsson A, Lalu MM, Li T, Loder EW, Mayo-Wilson E, McDonald S, McGuinness LA, Stewart LA, Thomas J, Tricco AC, Welch VA, Whiting P, Moher D (2021). The PRISMA 2020 statement: an updated guideline for reporting systematic reviews. BMJ.

[31] Geuens J, Swinnen TW, Westhovens R, de Vlam K, Geurts L, Abeele VV (2016). A review of persuasive principles in mobile apps for chronic arthritis patients: opportunities for improvement. JMIR Mhealth Uhealth.

[32] Machado GC, Pinheiro MB, Lee H, Ahmed OH, Hendrick P, Williams C, Kamper SJ (2016). Smartphone apps for the self-management of low back pain: a systematic review. Best Pract Res Clin Rheumatol.

[33] Wood CE, Richardson M, Johnston M, Abraham C, Francis J, Hardeman W, Michie S (2015). Applying the behaviour change technique (BCT) taxonomy v1: a study of coder training. Transl Behav Med.

[34] Blanquero J, Cortés-Vega MD, Rodríguez-Sánchez-Laulhé P, Corrales-Serra B, Gómez-Patricio E, Díaz-Matas N, Suero-Pineda A (2020). Feedback-guided exercises performed on a tablet touchscreen improve return to work, function, strength and healthcare usage more than an exercise program prescribed on paper for people with wrist, hand or finger injuries: a randomised trial. J Physiother.

[35] Blanquero J, Cortés-Vega MD, García-Frasquet MA, Sánchez-Laulhé PR, de Los Bernardos MIND, Suero-Pineda A (2019). Exercises using a touchscreen tablet application improved functional ability more than an exercise program prescribed on paper in people after surgical carpal tunnel release: a randomised trial. J Physiother.

[36] Keel S, Schmid A, Keller F, Schoeb V (2023). Investigating the use of digital health tools in physiotherapy: facilitators and barriers. Physiother Theory Pract.

[37] Alkhaldi O, McMillan B, Maddah N, Ainsworth J (2023). Interventions aimed at enhancing health care providers' behavior toward the prescription of mobile health apps: systematic review. JMIR Mhealth Uhealth.

[38] Giebel GD, Speckemeier C, Abels C, Plescher F, Börchers K, Wasem J, Blase N, Neusser S (2023). Problems and barriers related to the use of digital health applications: scoping review. J Med Internet Res.

[39] Biørn-Hansen A, Grønli TM, Ghinea G, Alouneh S (2019). An empirical study of cross-platform mobile development in industry. Wirel Commun Mob Comput.

[40] Balikuddembe JK, Reinhardt JD (2020). Can digitization of health care help low-resourced countries provide better community-based rehabilitation services?. Phys Ther.

[41] Salazar A, de Sola H, Failde I, Moral-Munoz JA (2018). Measuring the quality of mobile apps for the management of pain: systematic search and evaluation using the mobile app rating scale. JMIR Mhealth Uhealth.

[42] Mani M, Kavanagh DJ, Hides L, Stoyanov SR (2015). Review and evaluation of mindfulness-based iPhone apps. JMIR Mhealth Uhealth.

[43] Wang C, Qi H (2021). Influencing factors of acceptance and use behavior of mobile health application users: systematic review. Healthcare (Basel).

[44] Handayani PW, Gelshirani NB, Azzahro F, Pinem AA, Hidayanto AN (2020). The influence of argument quality, source credibility, and health consciousness on satisfaction, use intention, and loyalty on mobile health application use. Inform Med Unlocked.

[45] Akbar S, Coiera E, Magrabi F (2020). Safety concerns with consumer-facing mobile health applications and their consequences: a scoping review. J Am Med Inform Assoc.

[46] Dona SWA, Nguyen D, Angeles MR, Cooper P, Winter N, Chatterton ML, Peeters A, Hensher M (2022). Developing and testing a health app evaluation framework for organisations to recommend the best health apps for consumers in the Australian setting: executive summary. Deakin University—Deakin Health Economics, School of Health and Social Development, Institute for Health Transformation.

[47] Terhorst Y, Philippi P, Sander LB, Schultchen D, Paganini S, Bardus M, Santo K, Knitza J, Machado GC, Schoeppe S, Bauereiß N, Portenhauser A, Domhardt M, Walter B, Krusche M, Baumeister H, Messner EM (2020). Validation of the Mobile Application Rating Scale (MARS). PLoS One.

[48] Nelligan RK, Hinman RS, Kasza J, Crofts SJC, Bennell KL (2021). Effects of a self-directed web-based strengthening exercise and physical activity program supported by automated text messages for people with knee osteoarthritis: a randomized clinical trial. JAMA Intern Med.

[49] Bennell K, Nelligan RK, Schwartz S, Kasza J, Kimp A, Crofts SJ, Hinman RS (2020). Behavior change text messages for home exercise adherence in knee osteoarthritis: randomized trial. J Med Internet Res.

[50] Hinman RS, Nelligan RK, Campbell PK, Kimp AJ, Graham B, Merolli M, McManus F, Lamb KE, Bennell KL (2022). Exercise adherence mobile app for knee osteoarthritis: protocol for the MappKO randomised controlled trial. BMC Musculoskelet Disord.

[51] Merolli M, Hinman RS, Lawford BJ, Choo D, Gray K (2021). Digital health interventions in physiotherapy: development of client and health care provider survey instruments. JMIR Res Protoc.

[52] Zanaboni P, Ngangue P, Mbemba GIC, Schopf TR, Bergmo TS, Gagnon MP (2018). Methods to evaluate the effects of internet-based digital health interventions for citizens: systematic review of reviews. J Med Internet Res.

[53] Blandford A, Gibbs J, Newhouse N, Perski O, Singh A, Murray E (2018). Seven lessons for interdisciplinary research on interactive digital health interventions. Digit Health.

[54] Kunstler BE, Cook JL, Freene N, Finch CF, Kemp JL, O'Halloran PD, Gaida JE (2018). Physiotherapists use a small number of behaviour change techniques when promoting physical activity: a systematic review comparing experimental and observational studies. J Sci Med Sport.

[55] Söderlund A, Elvén M, Sandborgh M, Fritz J (2020). Implementing a behavioral medicine approach in physiotherapy for patients with musculoskeletal pain: a scoping review. Pain Rep.

[56] Hay-Smith EJC, McClurg D, Frawley H, Dean SG (2016). Exercise adherence: integrating theory, evidence and behaviour change techniques. Physiotherapy.

[57] Bassett SF (2015). Bridging the intention-behaviour gap with behaviour change strategies for physiotherapy rehabilitation non-adherence. N Z J Physiother.

[58] Arkkukangas M, Cederbom S, Tonkonogi M, Carlsson ÕU (2021). Older adults' experiences with mHealth for fall prevention exercise: usability and promotion of behavior change strategies. Physiother Theory Pract.

[59] Berry A, McCabe CS, Muir S, Walsh N (2018). Digital behaviour change interventions to facilitate physical activity in osteoarthritis: a systematic review. Phys Ther Rev.

[60] Beresford L, Norwood T (2022). The effect of mobile care delivery on clinically meaningful outcomes, satisfaction, and engagement among physical therapy patients: observational retrospective study. JMIR Rehabil Assist Technol.

[61] Stoyanov SR, Hides L, Kavanagh DJ, Wilson H (2016). Development and validation of the user version of the Mobile Application Rating Scale (uMARS). JMIR Mhealth Uhealth.

[62] Vaghefi I, Tulu B (2019). The continued use of mobile health apps: insights from a longitudinal study. JMIR Mhealth Uhealth.

