# Supplementary material for: Evaluation of Patient-Facing Mobile Apps to Support Physiotherapy Care: Systematic Review
Source: JMIR Mhealth Uhealth. 2024 Mar 4;12:e55003. doi: 10.2196/55003 (PMC10949126; doi:10.2196/55003)
Supplement: Multimedia Appendix 2 [file mhealth_v12i1e55003_app2.pdf]

App name	Data privacy transp-arency	Companion app	Platform	App focus	Physio specialty	Targeted behaviors if evident:	Simplified target behaviors	Country (origin)	Developer qualifications	Google Play (app version)	Google Play Payment Method	Google Play cost (AUD$)	Apple app version)	Apple Payment method	Apple cost (AUD$)
A Rehab Diary	Y	-	Both	General	-	Perform training/rehabilitation exercise program, track movement, set goals, compliance, RPE and pain throughout program	Performing therapeutic exercise (general); Recording information about exercise; Communication with health professional	Australia	HCP	1.1	Free	Free	-	Free	Free
AllyCare	Y	Y	Both	General	MSK	Perform rehabilitation exercises; communicate with health professional; track progress; set goals	Performing therapeutic exercise (general); Recording information about exercise; Communication with health professional; setting goals	Singapore	Not clear	1.4.11	Free (in app purchases available)	Free	1.4.11	Free (subscription version also available)	Free
Back Pain Diary	Y	-	Both	Back pain	MSK		Recording information about exercise	Netherlands	Not clear	1.0.3	In-app purchases	$4.89 - $32.99 / item	1.1	Free (subscription version also available)	Free
BlueJay Engage - Patient	Y	Y	Both	General	-	Perform home exercise program; communicate with your therapist; log pain and movement progress	Performing therapeutic exercise (general); Recording information about exercise; Recording information about health condition; Communication with health professional	United States	Not clear	8.1.7	Free	Free	8.1.7	Free	Free
Complex Core	Y	Y	Both	Core, arm, legs	MSK	Perform flexibility and strength training, and coordination	Performing therapeutic exercise (general)	Austria	Not clear	1.2.0	One off payment	$8.99	2.0.0	One off payment	$9.99

						and endurance training									
CP-Fit	Y	-	Apple	Cerebal palsy	Paeds	Perform exercises	Performing therapeutic exercise (paediatrics-cerebral palsy); Recording information about exercise	United States	Not clear	-	-	-	-	Free	Free
Embodia	Y	Y	Both	General	-	Access and perform your personalised home exercise program; track your adherence and progress; securely communication with your practitioner	Performing therapeutic exercise (general); Recording information about exercise; Communication with health professional	Not clear	Not clear	3.3.0	Free	Free	4.3.0	Free	Free
ExorLive Go	Y	Y	Both	General	MSK	Perform training/rehabilitation exercise program; track movement, set goals, compliance, progress	Performing therapeutic exercise (general); Recording information about exercise; Communication with health professional	Norway	HCP	4.0.3	Free	Free	4.0.3	Free (in app purchases available)	Free
Extensor -- Physio Patients	Y	Y	Both	General	-	shows you how to perform your exercises; remember how to do your exercises within app reminders	Performing therapeutic exercise (general); Recording information about exercise; Communication with health professional	Austria	Not clear	1.2.1	Free	Free	1.2.5	Free (subscription version also available)	Free

Guided Physio	Y	-	Both	General	MSK	Patients better managing their conditions with or without the assistance of healthcare professionals; work through pre-determined programs for the patients particular condition	Performing therapeutic exercise (general)	Not clear	HCP	1.9.5	One off payment	$4.99	1.9.4	One off payment	$4.49
Haem Active	Y	-	Both	Haemop hilia	-	Complete personalised exercise plan (on own or in consultation with physiotherapist); evaluate how you feel before and after exercise; set reminders to perform exercises	Performing therapeutic exercise (haemophilia); Recording information about exercise	Unclear	HCP	1.3.0	Free	Free	1.3.4	Free	Free
Home Physio	N	-	Both	General	-	Perform exercise plan	Performing therapeutic exercise (general); Recording information about exercise	Not clear	Not clear	1.9.41	Free	Free	1.9.31	Free	Free
My Exercise Messages	Y	-	Both	Osteoa-rthritis (lower limb)	MSK	Setting exercise goals, recording & monitoring of completed exercise sessions, inputting barriers to adherence & receipt of messages to overcome exercise barriers	Recording information about exercise; setting goals, education	Australia	HCP	1.0.7	Free	Free	1.0.5	Free	Free

My Exercise Program	Y	-	Both	General	-	complete exercise program; log your symptoms and progress	Performing therapeutic exercise (general); Recording information about exercise; Communication with health professional	Australia	HCP	1.0.4	Free	Free	1.0.4	Free	Free
My Injury	N	-	Apple	General		Perform strengthening and flexibility exercises for injury recovery; perform taping techniques; read tips for recovery	Performing therapeutic exercise (general); General self-care	Unclear	Not clear	-	-	-	1.6	One off payment	$1.49
OT App Lite	Y	-	Apple	General	Paeds/ MSK	Perform exercises in flash-card form, including full body movements that focus on shoulder, wrist, elbow and finger movement	Performing therapeutic exercise (paediatrics)	United States	Not clear	-	-	-	1.1	Free	Free
PhysiApp	Y	Y	Both	General	-	Shows you how to perform your exercises; remember how to do your exercises within app reminders	Performing therapeutic exercise (general); Recording information about exercise; Communication with health professional	United Kingdom	Not clear	4.6.1	Free	Free	2.2.7	Free	Free
Physiotools Trainer	Y	Y	Both	General	-	Motivates and reminds you to complete the exercises; set up automatic reminders and preview upcoming exercises to plan your schedule; supports you in	Performing therapeutic exercise (general); Recording information about exercise	Not clear	Not clear	1.0.919	Free	Free	1.0.904	Free	Free

						reaching your rehabilitation and fitness goals									
Pocket Physio	N	-	Apple	General	-	Perform strengthening and flexibility exercises; perform taping techniques; engage in injury rehabilitation information	Performing therapeutic exercise (peri-operative); Post-surgical self-care	Not clear	HCP	-	-	-	2.0.4	One off payment	$1.49
PT Timer: Stretch & Exercise	Y	-	Apple	General	-	Perform exercises; schedule your exercises and receive reminders	Performing therapeutic exercise (general); Recording information about exercise	Not clear	Not clear	-	-	-	3.3.12	Free (in app purchases available)	Free
PT-Helper Pro	Y	Y	Both	General	MSK	Perform home exercise program (focusing on fitness/wellness exercises for the ankle, back, elbow, fingers, hip, knee, neck, shoulder and wrist); set daily reminders	Performing therapeutic exercise (general); Recording information about exercise	United States	Non-HCP	2.5.3	One off payment	$4.89	1.3.0	One off payment	$4.49
RecovAware Knee Health Fitness	Y	-	Apple	Knee	MSK	Self-management of ongoing knee conditions (arthritis, patellofemoral pain etc)	Performing therapeutic exercise (knee); Recording information about exercise; Recording information about health condition;	Unclear	HCP	-	-	-	2.1.4	Free (in app purchases available)	Free

							Connect with friends; General self-care; Post-surgical self-care							
Rehab Guru Client	Y	Y	Both	General	-	View your exercise training program; provide feedback on pain and wellness; track your progress; set reminders to perform your exercises	Performing therapeutic exercise (general); Recording information about exercise; Recording information about health condition	United Kingdom	HCP	3.0.5	Free	Free	3.0.3	Free	Free
Rehand, Hand Rehabilitation	Y	-	Both	Wrist, hand, finger	-	Perform exercise program prescribed for rehabilitation	Performing therapeutic exercise (hand therapy); Recording information about exercise; Communication with health professional	Spain	HCP	3.4.5	In-app purchases	$50.99 per item	3.4.5	Free	Free
Smart Therapist	N	-	Apple	General	-	Follow the advice of your therapist or trainer using recordings made during the appointment, review and recall how the exercise should be done; record the level of pain felt, number of repetitions, resistance and more	Performing therapeutic exercise (general); Recording information about exercise; Recording information about health condition; Connect with health professional (make appointment)	Australia	Non-HCP	-	-	-	2.3.0	Free	Free

Squeezy	Y	-	Both	Pelvic health	Pelvic health (Wome n's)	Help women to remember to do their pelvic floor muscle exercises	Performing therapeutic exercise (women's health); Recording information about exercise; Recording information about health condition	United Kingdom	HCP	4.2.1	One off payment	$4.99	2.7.1	One off payment	$4.49
Squeezy for Men	Y	-	Both	Pelvic health	Pelvic health (Men's)	Support pelvic floor muscle exercise program; set to remind you when to do your exercises	Performing therapeutic exercise (men's health); Recording information about exercise	United Kingdom	HCP	4.2.1	One off payment	$4.99	4.0.0	One off payment	$4.49
Squeezy: CF	Y	-	Apple	Pelvic health (cyctic fibrosis)	Pelvic health	Support pelvic floor muscle exercise program; set to remind you when to do your exercises	Performing therapeutic exercise (pelvic floor in cystic fibrosis); Recording information about exercise	United Kingdom	HCP	1.2.11	One off payment	$4.99	1.0.1	One off payment	$4.99
Switchback Health	Y	Y	Apple	General	-	Perform exercises prescribed by your physical therapist	Performing therapeutic exercise (general); Recording information about exercise	United States	HCP	-	-	-	1.4	Free	Free
TeleHab	Y	Y	Both	General	-	Perform training/rehabilitation exercise program; track movement compliance, RPE and pain throughout program	Performing therapeutic exercise (general); Recording information about exercise; Communication with health professional	Australia	HCP	1.2	Free	Free	2.2.6	Free	Free
Track Rehab	Y	Y	Apple	General	MSK	Perform rehabilitation exercises; communicate with physiotherapist	Performing therapeutic exercise (general); Recording information about exercise; Communication	United Kingdom	HCP	-	-	-	-	Free (subscription version also available)	Free

							with health professional								
TrackActive Pro - Patient App	Y	Y	Both	General	-	Complete exercise programs; log your symptoms and progress	Performing therapeutic exercise (general); Recording information about exercise; Connect with health professional (make appointment)	Not clear	Not clear	1.1.5	Free	Free	1.8.2	Free	Free
Vrsteps Home rehabilitation	Y	Y	Google play	General	MSK	Perform rehabilitation exercises; communicate with health professional, track progress	Performing therapeutic exercise (general); Recording information about exercise; Communication with health professional	Isreal	HCP	0.975	Free (subscripti on version also available)	Free	-	-	-
Wheelchair Exercises	Y	-	Apple	Wheelc hair	Paeds/ MSK	Perform exercises in flash-card form, including some full body movements	Performing therapeutic exercise (paediatrics)	United States	Not clear	2	One off payment	$7.49	-	One off payment	$7.99
YRMOVE	Y	-	Apple	General	MSK	Perform rehabilitation exercises; communicate with physiotherapist	Performing therapeutic exercise (general); Recording information about exercise	Not clear	Not clear	-	-	-	1.0.6	Free (subscription version also available)	Free

Abbreviations: HCP = Healthcare Professional; MSK = Musculoskeletal; Paeds = Paediatrics
