# Supplementary material for: Evaluation of Patient-Facing Mobile Apps to Support Physiotherapy Care: Systematic Review
Source: JMIR Mhealth Uhealth. 2024 Mar 4;12:e55003. doi: 10.2196/55003 (PMC10949126; doi:10.2196/55003)
Supplement: Multimedia Appendix 3 [file mhealth_v12i1e55003_app3.pdf]

ABACUS item and description	Scoring system
**1. Knowledge and information**	
1.1 Does the app have the ability to customize and personalize some features?	Score if app scored >1/5 on MARS section A.3
1.2 Was the app created with expertise and/or does the app provide information that is consistent with national guidelines?	Score if mention of relevant expertise (i.e. health care professional) involved in app creation in app store or within app, or if BCT 9.1, credible source, is present.
1.3 Does the app ask for baseline information?	
1.4 Does the app provide instruction on how to perform the behavior?	Score if BCT 4.1, Instruction on how to perform behavior, or BCT 6.1, demonstration of behavior, is present.
1.5 Does the app provide information about the consequences of continuing and/or discontinuing behavior?	Score if BCT 5.1, Information about health consequences, BCT 5.2, salience of consequences, BCT 5.6, Information about emotional consequences or BCT 5.3, Information about social and environmental consequences, is present.
**2. Goals and planning**	
2.1 Does the app ask for willingness for behavior change?	
2.2 Does the app allow for the setting of goals?	Score if BCT 1.1, Goal setting (behavior), or BCT 1.3, Goal setting (outcome), is present.
2.3 Does the app have the ability to review goals, update, and change when necessary?	Score if BCT 1.5, Review behavior goal(s), or BCT 1.7, Review outcome goals, is present.
**3. Feedback and monitoring**	
3.1 Does the app give the user the ability to quickly and easily understand the difference between current action and future goals?	Score if BCT 1.6, Discrepancy between current behavior and goals, is present.
3.2 Does the app have the ability to allow the user to easily self-monitor behavior?	Score if BCT 2.3, Self-monitoring of behavior, or BCT 2.4, Self-monitoring of outcome(s) of behavior, is present.
3.3 Does the app have the ability to share behaviors with others (including social media	Score if BCT 3.1, Social support (unspecified), BCT 3.2, Social support (practical), or BCT 3.3, Social support (emotional), is present.

or forums) and/or allow for social comparison?	
3.4 Does the app have the ability to give the user feedback—either from a person or automatically?	Score if BCT 2.2, Feedback on behavior, or BCT 2.7, Feedback on outcome(s) of behavior, or BCT 2.6, Biofeedback, is present.
3.5 Does the app have the ability to export data from app?	Score if app has ability to "deliberately" export data.
3.6 Does the app provide a material or social reward or incentive?	Score if BCT 10.1, Material incentive (behavior), or BCT 10.2, Material reward (behavior), or BCT 10.4, Social reward, or BCT 10.5 Social incentive, is present.
3.7 Does the app provide general encouragement?	Score if BCT 10.3, Non-specific reward, or BCT 10.6, Non-specific incentive, is present.
**4. Actions**	
4.1 Does the app have reminders and/or prompts or cues for activity?	Score if BCT 7.1, Prompts/cues, is present.
4.2 Does the app encourage positive habit formation?	Score if BCT 8.1, Behavioral practice/rehearsal, is present.
4.3 Does the app allow or encourage for practice or rehearsal, in addition to daily activities?	Don't score if app 'locks' user access (including viewing) to content after reaching daily limit, otherwise score.
4.4 Does the app provide opportunity to plan for barriers?	Score if BCT 1.2, Problem solving, or 1.4, Action planning, is present.
4.5 Does the app assist with or suggest restructuring the physical or social environment?	Score if BCT 12.1, Restructuring the physical environment, or BCT 12.2, Restructuring the social environment, is present.
4.6 Does the app assists with distraction or avoidance?	Score if BCT 12.3, Avoidance/ reducing exposure to cues for behavior, or BCT 12.4, Distraction, is present.
