# Supplementary material for: Evaluation of Patient-Facing Mobile Apps to Support Physiotherapy Care: Systematic Review
Source: JMIR Mhealth Uhealth. 2024 Mar 4;12:e55003. doi: 10.2196/55003 (PMC10949126; doi:10.2196/55003)
Supplement: Multimedia Appendix 4 [file mhealth_v12i1e55003_app4.docx]

| **App and BCT** | **1.1** | **1.2** | **1.3** | **1.4** | **1.5** | **1.6** | **1.7** | **2.1** | **2.2** | **2.3** | **2.4** | **2.5** | **2.6** | **2.7** | **3.1** | **3.3** | **4.1** | **5.1** | **6.1** | **6.2** | **7.1** | **8.1** | **8.3** | **9.1** | **9.2** | **10.3** | **10.4** | **10.6** |  |
| --- | --- | --- | --- | --- | --- | --- | --- | --- | --- | --- | --- | --- | --- | --- | --- | --- | --- | --- | --- | --- | --- | --- | --- | --- | --- | --- | --- | --- | --- |
| A Rehab Diary |  | 1 | 1 | 1 | 1 | 1 | 1 |  | 1 | 1 |  |  |  | 1 | 1 |  | 1 |  |  |  | 1 |  |  | 1 |  |  |  |  | **13** |
| AllyCare | 1 |  |  | 1 | 1 |  |  | 1 | 1 | 1 | 1 | 1 |  | 1 | 1 |  | 1 |  | 1 |  | 1 | 1 |  | 1 |  | 1 |  |  | **16** |
| Back Pain Diary |  |  |  |  |  |  |  | 1 |  | 1 | 1 | 1 |  | 1 |  |  |  |  |  |  |  |  |  |  |  |  |  |  | **5** |
| BlueJay Engage - Patient | 1 |  |  | 1 |  |  |  | 1 | 1 | 1 | 1 | 1 |  | 1 | 1 |  | 1 |  | 1 |  | 1 |  |  | 1 |  |  |  |  | **13** |
| ComplexCore |  |  |  | 1 |  |  |  |  |  |  |  |  |  |  |  |  | 1 |  | 1 |  |  |  |  | 1 |  |  |  |  | **4** |
| CP-Fit |  |  |  | 1 |  |  |  |  | 1 | 1 |  |  |  |  |  |  | 1 |  | 1 |  |  |  |  |  |  | 1 |  |  | **6** |
| Embodia |  |  |  | 1 |  |  |  | 1 | 1 | 1 |  | 1 |  | 1 | 1 |  | 1 | 1 | 1 |  | 1 |  |  | 1 |  |  |  |  | **12** |
| ExorLive Go |  |  |  | 1 |  | 1 |  |  | 1 | 1 | 1 |  |  |  | 1 |  | 1 |  | 1 |  | 1 |  |  | 1 |  | 1 |  | 1 | **12** |
| Extensor- Physio Patients |  |  |  | 1 |  |  |  |  | 1 | 1 |  |  |  |  | 1 |  | 1 |  | 1 |  |  |  |  | 1 |  |  |  |  | **7** |
| Guided Physio |  |  |  |  |  |  |  |  | 1 | 1 |  |  |  |  |  |  | 1 | 1 | 1 |  |  |  |  | 1 |  |  |  |  | **6** |
| HaemActive |  |  |  | 1 |  |  |  |  | 1 | 1 | 1 |  |  | 1 |  |  | 1 | 1 | 1 |  | 1 |  |  | 1 |  |  |  |  | **10** |
| Home Physio |  |  |  | 1 |  |  |  |  |  |  |  |  |  |  |  |  | 1 |  | 1 |  | 1 |  |  |  |  |  |  |  | **4** |
| My Exercise Messages | 1 |  |  | 1 |  | 1 |  | 1 | 1 | 1 |  |  |  |  | 1 | 1 |  | 1 |  |  | 1 |  | 1 | 1 | 1 | 1 |  |  | **14** |
| My Exercise Program |  |  |  | 1 |  |  |  |  | 1 | 1 |  | 1 |  | 1 |  |  | 1 |  | 1 |  |  |  |  | 1 |  |  |  |  | **8** |
| My Injury |  |  |  |  |  |  |  |  |  |  |  |  |  |  |  |  | 1 | 1 | 1 |  |  |  |  |  |  |  |  |  | **3** |
| OT App Lite |  |  |  |  |  |  |  |  |  |  |  |  |  |  |  |  | 1 |  | 1 |  |  |  |  | 1 |  |  |  |  | **3** |
| PhysiApp |  |  |  | 1 |  |  |  | 1 | 1 | 1 | 1 | 1 |  | 1 | 1 |  | 1 | 1 | 1 |  | 1 |  |  | 1 |  |  |  |  | **13** |
| Physiotools Trainer |  |  |  | 1 |  |  |  |  | 1 | 1 |  |  |  |  |  |  | 1 |  | 1 |  | 1 |  |  |  |  |  |  |  | **6** |
| Pocket Physio |  |  |  | 1 |  |  |  |  |  |  |  |  |  |  |  |  | 1 | 1 | 1 |  | 1 |  |  | 1 |  |  |  |  | **6** |
| PT Timer: Stretch & Exercise |  |  |  | 1 |  |  |  |  | 1 | 1 |  |  |  |  |  |  | 1 |  |  |  | 1 |  |  |  |  |  |  |  | **5** |
| PT-Helper Pro |  |  |  | 1 |  |  |  | 1 | 1 | 1 |  |  |  |  |  |  | 1 |  | 1 |  | 1 |  |  | 1 |  |  |  |  | **8** |
| Recov  Aware Knee Health Fitness |  |  |  | 1 |  |  |  |  | 1 | 1 | 1 |  |  | 1 | 1 |  |  |  |  | 1 | 1 |  |  | 1 |  |  |  |  | **9** |
| Rehab Guru Client |  |  |  | 1 |  |  |  |  | 1 | 1 | 1 |  |  | 1 | 1 |  | 1 |  | 1 |  | 1 |  |  |  |  |  |  |  | **9** |
| Rehand, Hand Rehabilitation |  |  |  | 1 |  |  |  | 1 | 1 | 1 | 1 | 1 |  | 1 |  |  | 1 |  | 1 |  | 1 | 1 |  | 1 |  | 1 | 1 |  | **14** |
| Smart Therapist |  |  |  | 1 |  |  |  |  | 1 | 1 | 1 |  |  | 1 |  |  | 1 |  | 1 |  | 1 |  |  |  |  |  |  |  | **8** |
| Squeezy: CF |  |  |  | 1 |  |  |  |  | 1 | 1 |  |  |  |  |  |  | 1 | 1 |  |  | 1 |  |  | 1 |  |  |  |  | **7** |
| Squeezy for Men |  |  |  | 1 |  |  |  |  | 1 | 1 |  |  |  |  |  |  | 1 | 1 |  |  | 1 |  |  | 1 |  |  |  |  | **7** |
| Squeezy |  |  |  | 1 |  |  |  |  | 1 | 1 | 1 |  |  | 1 |  |  | 1 | 1 |  |  | 1 |  |  | 1 |  |  |  |  | **9** |
| Switchback Health |  |  |  | 1 |  |  |  | 1 | 1 | 1 |  |  |  |  | 1 |  | 1 |  | 1 |  | 1 |  |  |  |  |  |  |  | **8** |
| TeleHab |  |  |  | 1 |  |  |  | 1 | 1 | 1 | 1 |  |  | 1 | 1 |  | 1 | 1 | 1 |  | 1 |  |  | 1 |  |  | 1 |  | **13** |
| Track Rehab |  |  |  | 1 |  |  |  |  | 1 | 1 | 1 |  |  | 1 | 1 |  | 1 | 1 | 1 |  | 1 |  |  | 1 |  |  |  |  | **11** |
| Track  Active Pro - Patient App |  |  |  | 1 |  |  |  | 1 | 1 | 1 | 1 | 1 |  | 1 |  |  | 1 |  | 1 |  | 1 |  |  |  |  |  |  |  | **10** |
| Vrsteps Home Rehabilitation |  |  |  | 1 |  |  |  |  | 1 | 1 |  |  | 1 | 1 | 1 |  | 1 |  | 1 |  | 1 |  |  | 1 |  |  |  |  | **10** |
| Wheelchair Exercises |  |  |  |  |  |  |  |  |  |  |  |  |  |  |  |  | 1 |  | 1 |  |  |  |  | 1 |  |  |  |  | **3** |
| YRMOVE |  |  |  | 1 |  |  |  | 1 |  |  |  |  |  |  |  |  | 1 |  | 1 |  |  |  |  | 1 |  |  |  |  | **5** |
| **Total frequency of each BCT** | **3** | **1** | **1** | **30** | **2** | **3** | **1** | **12** | **27** | **28** | **14** | **8** | **1** | **17** | **14** | **1** | **32** | **12** | **27** | **1** | **25** | **2** | **1** | **25** | **1** | **5** | **2** | **1** | **8.5**  **(SD 3.6)** |

Coded BCTs included in each app (refer to Multimedia Appendix 5 for the glossary explaining each BCT)
