# Supplementary material for: Evaluation of Patient-Facing Mobile Apps to Support Physiotherapy Care: Systematic Review
Source: JMIR Mhealth Uhealth. 2024 Mar 4;12:e55003. doi: 10.2196/55003 (PMC10949126; doi:10.2196/55003)
Supplement: Multimedia Appendix 5 [file mhealth_v12i1e55003_app5.pdf]

App and BCT	1.1	1.2	1.3	1.4	1.5	1.6	1.7	2.1	2.2	2.3	2.4	2.5	2.6	2.7	3.1	3.3	4.1	5.1	6.1	6.2	7.1	8.1	8.3	9.1	9.2	10.3	10.4	10.6	
A Rehab Diary		1	1	1	1	1	1		1	1				1	1		1				1			1					**13**
AllyCare	1			1	1			1	1	1	1	1		1	1		1		1		1	1		1		1			**16**
Back Pain Diary								1		1	1	1		1															**5**
BlueJay Engage - Patient	1			1				1	1	1	1	1		1	1		1		1		1			1					**13**
ComplexCore				1													1		1					1					**4**
CP-Fit				1					1	1							1		1							1			**6**
Embodia				1				1	1	1		1		1	1		1	1	1		1			1					**12**
ExorLive Go				1		1			1	1	1				1		1		1		1			1		1		1	**12**
Extensor- Physio Patients				1					1	1					1		1		1					1					**7**
Guided Physio									1	1							1	1	1					1					**6**
HaemActive				1					1	1	1			1			1	1	1		1			1					**10**
Home Physio				1													1		1		1								**4**
My Exercise Messages	1			1		1		1	1	1					1	1		1			1		1	1	1	1			**14**
My Exercise Program				1					1	1		1		1			1		1					1					**8**
My Injury																	1	1	1										**3**
OT App Lite																	1		1					1					**3**
PhysiApp				1				1	1	1	1	1		1	1		1	1	1		1			1					**13**
Physiotools Trainer				1					1	1							1		1		1								**6**
Pocket Physio				1													1	1	1		1			1					**6**
PT Timer: Stretch & Exercise				1					1	1							1				1								**5**
PT-Helper Pro				1				1	1	1							1		1		1			1					**8**
Recov<br>Aware Knee Health Fitness				1					1	1	1			1	1					1	1			1					**9**

Rehab Guru Client				1					1	1	1			1	1		1		1		1								**9**
Rehand, Hand Rehabilitation				1				1	1	1	1	1		1			1		1		1	1		1		1	1		**14**
Smart Therapist				1					1	1	1			1			1		1		1								**8**
Squeezy: CF				1					1	1							1	1			1			1					**7**
Squeezy for Men				1					1	1							1	1			1			1					**7**
Squeezy				1					1	1	1			1			1	1			1			1					**9**
Switchback Health				1				1	1	1					1		1		1		1								**8**
TeleHab				1				1	1	1	1			1	1		1	1	1		1			1			1		**13**
Track Rehab				1					1	1	1			1	1		1	1	1		1			1					**11**
Track<br>Active Pro - Patient App				1				1	1	1	1	1		1			1		1		1								**10**
Vrsteps Home Rehabilitation				1					1	1			1	1	1		1		1		1			1					**10**
Wheelchair Exercises																	1		1					1					**3**
YRMOVE				1				1									1		1					1					**5**
**Total frequency of each BCT**	**3**	**1**	**1**	**30**	**2**	**3**	**1**	**12**	**27**	**28**	**14**	**8**	**1**	**17**	**14**	**1**	**32**	**12**	**27**	**1**	**25**	**2**	**1**	**25**	**1**	**5**	**2**	**1**	**8.5**

<u>BCT</u>

1.1 = Goal setting (behavior)
1.2 = Problem solving
1.3 = Goal setting (outcome)
1.4 = Action planning
1.5 = Review behavior goal(s)
1.6 = Discrepancy between current behavior and goal
1.7 = Review outcome goal(s)
2.1 = Monitoring of behavior by others without feedback
2.2 = Feedback on behavior
2.3 = Self-monitoring of behavior
2.4 = Self-monitoring of outcome(s) of behavior

2.5 = Monitoring outcome(s) of behavior by others without feedback
2.6 = Biofeedback
2.7 = Feedback on outcome(s) of behavior
3.1 = Social support (unspecified)
3.3 = Social support (emotional)
4.1 = Instruction on how to perform a behavior
5.1 = Information about health consequences
6.1 = Demonstration of the behavior
6.2 = Social comparison
7.1 = Prompts/cues
8.1 = Behavioral practice/rehearsal
8.3 = Habit formation
9.1 = Credible source
9.2 = Pros and cons
10.3 = Non-specific reward
10.4 = Social reward
10.6 = Non-specific incentive
